# Immediate Outcomes of Transcatheter Closure versus Surgical Ligation of Patent Ductus Arteriosus in Children at the Cardiac Center of Ethiopia (2012-2022): A Comparative Cross-Sectional Study

**DOI:** 10.4314/ejhs.v34i5.6

**Published:** 2024-09

**Authors:** Mohammed Nasir Beshir, Muluken Ahmed

**Affiliations:** 1 Pediatric Cardiologist, Hawassa University, Hawassa; 2 Pediatrician, Arba Minch University, Arba Minch

**Keywords:** PDA, surgical ligation of PDA, Transcatheter closure of PDA, comparative study

## Abstract

**Background:**

Transcatheter closure and surgical ligation of patent ductus arteriosus (PDA) are management options for PDA that have not spontaneously closed. However, studies reported the presence of complications and residual shunts in both Transcatheter closure and surgical ligation of PDA by themselves. In this study, the immediate outcomes of transcatheter closure of PDA versus surgical ligation of PDA were compared.

**Methods:**

Comparative cross-sectional study conducted on children under 18 years of age who underwent transcatheter closure and surgical ligation of PDA at a cardiac center in Ethiopia from January 1, 2012, to January 1, 2022, by retrospectively reviewing the records from October 1, 2023, to November 1, 2023. 664 patients who underwent PDA closure, (n = 316) in the transcatheter closure group and (n = 348) patients in the surgical ligation group were included in this study.

**Result:**

The surgical ligation group patients were younger, and more proportion of patients had severe pulmonary hypertension. Overall complications were significantly higher with surgical ligation compared with transcatheter closure ((112 (35.4% vs 286 (71.9%)), p-value = 0.001)). Total mechanical ventilation time, intensive care unit(ICU) stay, and hospital stay were higher in the surgical ligation group with a p-value of 0.001 each.

**Conclusion:**

Transcatheter closure of PDA has lower overall complications and shorter mechanical ventilation time, lower ICU stay, and lower hospital stay. Given the lower number of overall complications, transcatheter closure of PDA has to be considered for selected patients.

## Introduction

Ductus arteriosus is an important fetal structure that developed from the sixth left embryonic arch and serves as a passage between the left pulmonary artery and the aortic arch to relieve the right ventricle's afterload during fetal life(1). Its birth prevalence ranges from 0.87 to 1 per 1,000 live births and accounts for 10% of congenital cardiac disease(1–3). In normal-term babies, spontaneous closure of ductus arteriosus was anticipated to occur within 48 hours in 90% of cases and within 72 hours in 100% of instances(4).

Depending mainly on its size, the ductus arteriosus may persist even into later childhood or into adulthood due to the need for numerous factors in its spontaneous closure (4). Patent ductus arteriosus (PDA) if it is hemodynamically significant, can lead to several complications, such as heart failure brought on by increased pulmonary over circulation, aneurysmal dilatation of PDA with a risk of rupture, and in the long-term pulmonary obstructive illness (Eisenmenger syndrome). Infective endarteritis may also occur as a complication of small PDA(5,6). There are hence indications for PDA closure before the onset of the aforementioned complications. The indications are the presence of symptoms and left heart chamber dilatation regardless of the presence of symptoms(6).

The two management options for PDA are transcatheter closure and surgical ligation. Although both management options have complications and variations in outcomes across centers, transcatheter closure(TC) of PDA is on the rise in the developed world while surgical ligation(SL) is on the decline(6,7).

In developing countries, where closure is typically carried out in late childhood and adolescence, there isn't many researches done that compare the complication and overall in-hospital outcome of TL vs. SC. The few studies done in Pakistan and Indonesia that compared the outcome difference in patients who were managed with TC vs. SL showed that TC has lower complication, mortality, and length of hospital stay(8,9).

However, as far as we are aware there are only a few studies done in Africa that compare the immediate outcome of TC vs. SL of PDA. Therefore, the purpose of this study is to compare the immediate outcomes of the two PDA closure techniques in patients who have undergone PDA closure in a cardiac center of Ethiopia.

## Methods and Materials

**Study area and period**: The Cardiac Center of Ethiopia (CCE) is located in Addis Ababa, the capital of Ethiopia. It has offered free care to cardiac patients ever since it was established in 2012. It also provides both adult and pediatric cardiac surgeries as well as both adult and pediatric interventional procedures. The facility has five wards with ten beds each, one OR, one CATH lab, and one ICU with five beds. At least 50 adult and child patients are seen daily in this facility's outpatient department. The facility performs three cardiac operations and three interventional procedures each week. The hospital currently has 20 nurses working there, along with four cardiac and cardiothoracic surgeons, three pediatric cardiologists, and two adult cardiologists. This study was carried out in CCE from October 1, 2023 to November 1, 2023.

**Study design**: Comparative cross-sectional study

**Source population**: The source population consisted of all pediatric patients with PDA under the age of 18 who underwent TC or SL of PDA in the cardiac center of Ethiopia.

**Study population**: All PDA patients under the age of 18 who underwent PDA closure (TC or SL) in the cardiac center of Ethiopia between January 2012 and January 2022 were included in the study population.

**Inclusion and exclusion criteria**: All PDA patients under the age of 18 who had PDA closure (TC or SL) performed at the cardiac center in Ethiopia between January 2012 and January 2022 were included in the study. On the other hand, syndromic patients, patients with additional congenital heart disease to PDA, and patients with incompletely documented patient outcomes were excluded from this study.

### Procedure

**Transcatheter closure**: Patient selection for device closure is based on weight greater than 6 kg and age greater than 1 year, per hospital protocol. One to three millimeters more than the duct's minimum diameter device size were used. Before transcatheter closure of the PDA, we obtained informed consent from the patient's parents, administered a prophylactic dose of intravenous ceftriaxone, used transthoracic echocardiography to confirm the diagnosis of PDA, and ruled out other associated congenital heart diseases and used general anesthesia for sedation. In all of our patients, the transcatheter procedure began with the femoral artery and vein being punctured, a 5Fr. femoral sheath being inserted, and then heparin being administered. A 5Fr. MPA2 catheter was inserted to measure the pressure in the right ventricle and pulmonary arteries. Aortography was performed after the descending aorta was catheterized with a 5 Fr. pigtail catheter. Images of the PDA were obtained using angiography using lateral and right lateral oblique views. The angiographic result guided the device choice in our setting. The delivery sheath was advanced using the femoral vein. In our center, ADO-1, ADO-2, Lifetech PDA device, Cocoon PDA device, PFM device, Amplatzer muscular VSD occluder, and coils were in use. Transthoracic echocardiography was performed to evaluate the remaining shunt after device deployment angiography. Following the procedure, the patients were monitored for complications for 12 hours in the hospital before discharge, and will be discharged receiving follow-up care two weeks later.

### Surgical ligation

Likewise, before surgical ligation of PDA, we obtained informed consent from the patient's parents, administered a prophylactic dose of intravenous ceftriaxone, and used transthoracic echocardiography to confirm the diagnosis of PDA, and to rule out other associated congenital heart defects. General anesthesia for sedation was used in all cases. After that, a posterolateral incision was made in the left chest, double ligation of PDA, or division and double ligation of PDA, or double ligation with clipping with metallic clip or clipping with metallic clip alone were performed based on surgeon preference. A prophylactic chest tube was inserted and the patients were monitored for complications overnight and discharged the next day after being given an appointment after a week.

### Study variables

**Dependent variables**: The difference in the immediate outcome of TC Vs SL of PDA

**Independent variable**: PDA closure types (TC or SL)

**Covariates**: Echocardiographic size of PDA, age at diagnosis, age at procedure, sex, weight at surgery, height at surgery, and presence of severe pulmonary hypertension

### Definitions and operational definitions

**Postoperative or postprocedural complications -** the presence of any cardiac or extracardiac complications postoperatively or post-procedure in the hospital.

**Small residual PDA-** residual PDA of <1.5mm

**Moderate residual PDA**-residual PDA of 1.5-3mm

**Large residual PDA**-residual PDA of >3mm

**Mild Left ventricular dysfunction-** Left ventricular ejection fraction of 40-49%

**Moderate left ventricular dysfunction-** Left ventricular ejection fraction of 30-39%

**Severe left ventricular dysfunction**-Left ventricular ejection fraction of less than 30%

**Small pericardial effusion**-the maximum echo-free space between the heart and pericardium of <10mm in subcostal and apical four-chamber view

**Moderate pericardial effusion-** the maximum echo-free space between the heart and pericardium of 10-20mm in subcostal and apical four-chamber view

**Large pericardial effusion-** the maximum echo-free space between the heart and pericardium of greater than >20mm in subcostal and apical four-chamber view

**Small pleural effusion**-the maximum echo-free space between the lung and dome of diaphragm <10mm in subcostal view.

**Moderate pleural effusion-** the maximum echo-free space between the lung and dome of the diaphragm 10-20mm in the subcostal view

**Large pleural effusion-** the maximum echo-free space between the lung and dome of the diaphragm of greater than >20mm in subcostal view.

**Flow acceleration in the aortic arch left pulmonary artery, and the right pulmonary artery-** doppler velocity greater than 2m/s in the aortic arch, left pulmonary artery, and right pulmonary artery respectively.

**Acute kidney injury**-absolute increase in serum creatinine of 0.3mg/dl or a 1.5-fold increase in serum creatinine from the baseline.

**Systemic hypertension**-blood pressure that was at or above the 95^th^ percentile for children who are the same sex, age, and height.

**Pulmonary hypertension**-measured in a study by subtracting the systolic gradient of PDA in CW from the systolic pressure of the patient to get the systolic pulmonary pressure.

**Pulmonary hypertension crisis**-in a patient who had/developed pulmonary hypertension later/postoperatively or post device closure) presented with tachycardia, hypotension, poor perfusion, altered mental status, and enlarged liver.

**Low cardiac output patients** who presented with altered mental status, cold extremity, tachycardia or bradycardia, hypotension, low urine output(<0.5ml/kg/hr.), and metabolic acidosis.

**Immediate Outcome-** measured in this study by the presence of any complications, length of hospital stays, the presence of residual shunts, and death.

**Mortality-** death occurrence at any time before discharge from the hospital after post-surgery or post-procedure.

**Data collection procedure and tools**: Data was collected for a period of 1 month from October 1, 2023 to November 1, 2023. A structured questionnaire was used for data extraction. The questionnaire was adopted from the Society of Thoracic Surgeon version 3.1 form. The author did not have access to information that could identify individual participants during or after data collection.

**Data quality assurance**: To ensure the internal validity (accuracy and precision) of the study, maximum effort was taken to ensure the quality of data, to minimize errors and bias using the following measures, two days training for data collectors and supervisors was carried out to have a clear understanding about the objective of the study and data collection procedure.

The pre-testing of the structured format will be conducted on a 5% sample at Tasma Hospital (a private Hospital in Addis Ababa), who undergone PDA closure for PDA patients, before data collection to assess the quality of the questionnaire. Process and structured format will be checked for completeness daily by immediate supervisors and the principal investigator. After checking for consistency and completeness, the supervisors submitted the filled questionnaire to the principal investigator who rechecked the questionnaire, to maintain the quality of data. Data was cleaned & and entered by the principal investigator and strict daily field supervision and spot-checking were carried out.

**Data analysis**: The accuracy of the data was manually verified. The data was then cleansed and saved for consistency after being entered into the Epi info 7 program. The data was once again exported for analysis using SPSS version 28 and Microsoft Excel 13. After verifying the normality of continuous variables, appropriate descriptive statistics were performed. To verify normality, the Shapiro-Wilk test was applied. Categorical variables were described by frequency and percentage. Multivariable logistic regression was used to generate propensity scores based on the baseline characteristics of the patients in the SL and TC groups. The propensity scores multivariate logistic regression model used in the primary analyses considered the following variables: echocardiographic size of PDA, age at diagnosis, age at procedure, sex, weight at surgery, height at surgery, and presence of severe pulmonary hypertension. Using the inverse probability of treatment weighting (IPTW) by the propensity score and after adjusting for baseline variables, the Man U test, Chi-square test, and Fisher's exact test were used to compare immediate outcomes of TC and SL of PDA. The statistical significance was assessed using a 2-tailed p-value <0.05.

**Ethical consideration**: Before beginning the actual research, the ethical review committee and advisor gave their approval for the study. Hawassa University granted its ethical approval. The medical director of CCE was informed of the study's objectives and data from patient charts were extracted without using any personal identifiers to ensure confidentiality, and it was solely utilized for this study purpose.

**Ethical considerations**: Written informed consent was obtained from a research ethics review board at Saint Paul Millennium College to extract data from patients' medical records.

## Results

**Socio-demographic characteristics**: The socio-demographic characteristics of both Groups, Group-TC, and Group-SL are presented in **Error! Reference source not found..** In this study, 316 patients (47.6%) had TC, while 348 patients (52.4%) had SL of PDA. At the time of surgery, the median age for Group 1-TC was 3.8 (IQR: 1.6–5.6) and for Group 2-SC, it was 3 (IQR: 1.7–6; P = 0.15). Females made up 178(56.3%) in Group-TC and 213(61.8%) in Group-SL. Age at diagnosis, weight at surgery, and height at surgery, presence of severe PAH before surgery showed statistical differences between the two groups (TC and SC) with P values of 0.04, 0.02, 0.002, and 0.001 respectively.

**Figure 1:**
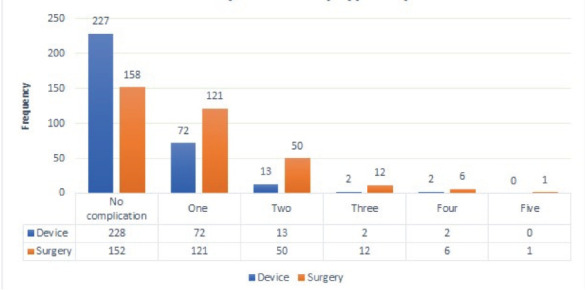
Demonstrating the number of complications of TC and SL of PDA in patients who have undergone PDA closure in the Cardiac Center of Ethiopia, 2012-2022

**Complications of PDA closure**: Table 2 demonstrates the two groups' percentages of various complications (SL group and TC group). Patients in Group SL had higher overall complications compared to Group TC. The SL group had a higher magnitude of all complications except flow acceleration in the RPA, LPA, and descending aorta. Encroachment of the device into the LPA and descending aorta caused flow acceleration in those vessels, whereas embolization of the device into the RPA caused flow acceleration in that vessel. (Table2, Figure 1)

**Table 1:** Presents the socio-demographic and basic characteristics of patients who underwent PDA closure at the Cardiac Center of Ethiopia, 2012-2022

Variables		TC	SC	P value
		N=316 (47.6%)	N=348 (52.4%)	
Age at diagnosis, median (IQR) (year)		3(1.6-5.6)	2.4(1-5)	0.04
Age at procedure, median (IQR)(year)		3.8(1.6- 6)	3(1.7-6)	0.15
Sex				0.20
	Male n (%)	138(43.7)	135(38.8)	
	Female n (%)	178(56.3)	213(61.8)	
Weight at surgery, median (IQR) (Kg)		12(10-16)	11(10-16)	0.02
Height at surgery, median (IQR)(cm)		100(92-107)	98(83-107)	0.002
Echocardiographic size of PDA, median (IQR) (mm)		5(4-8)	6(4-7)	0.06
Angiographic size of PDA, median (IQR)(mm)		4.5(3.5-7)	NA	-
Severe pulmonary hypertension				0.001
	Yes n (%)	96(30.4)	184(52.9)	
	No n (%)	220(69.6)	164(47.1)	

**Table 2:** shows a comparison of complications of patients who underwent PDA closure in the cardiac center of Ethiopia, 2012-2022

Complications	Group-TC 316(47.6%)	Group-SL 348 (52.4%)	P value
Mild LV dysfunction	14(4.4)	34(9.8)	0.001
Moderate LV dysfunction	12(3.8)	18(5.2)	0.62
Severe LV dysfunction	2(0.6%)	6(1.7)	0.99
small pericardial effusion	2(0.6)	8(2.3)	0.01
Moderate pericardial effusion	0(0)	2(0.6)	0.99
Moderate pleural effusion	0(0)	2(0.6)	0.99
Pneumothorax	2(0.6)	2(0.6)	0.34
Chylothorax	0(0)	4(1.1)	0.99
Hemothorax	0(0)	4(1.1)	0.99
Flow acceleration in the LPA	6(1.9)	4(1.1)	0.36
Flow acceleration in the RPA	6(1.9)	4(1.1)	0.03

Table 1: Continued…			

Flow acceleration over the aortic arch	8(2.5)	4(1.1)	0.01
Ascending aorta to RPA fistula	0(0)	2(0.6)	0.99
Hypertension	6(1.9)	40(11.5)	0.001
Pulmonary hypertension crisis	0(0)	2(0.6)	0.99
Hyperkalemia	0(0)	2(0.6)	0.99
Hypokalemia	0(0)	8(2.3)	0.99
Hyperglycemia	0(0)	6(1.7)	0.98
Bleeding that needs a transfusion	7(2.2)	12(3.4)	0.13
Sepsis	0(0)	2(0.6)	0.99
Acute kidney injury	0(0)	8(2.3)	0.99
Metabolic acidosis	0(0)	10(2.9)	0.99
Metabolic alkalosis	0(0)	2(0.6)	0.99
Thrombocytopenia	0(0)	2(0.6)	0.99
Low cardiac output	0(0)	4(1.1)	0.99
Femoral pulse loss	2(0.6)	0(0)	0.99
Chest wall hematoma	0(0)	2(0.6)	0.99
Atelectasis	4(1.3)	8(2.3)	0.30
Paraplegia	0(0)	4(1.1)	0.98
One complication n (%)	72(37.3)	121(62.7)	0.001
Two complications	14(8.8)	50(79.4)	
Three or more complications	4(3.8)	19(82.6)	
Overall complication n (%)	112(35.4)	286(71.9)	0.001
Opioid use	3(0.94)	55(15.8)	0.001

**Comparison between TC and SC outcome**: Differences in total length of mechanical ventilation, length of ICU stay, and length of hospital stay were statistically significant between Group-TC and Group-SC patients, all stays were more in Group-SC patients. There was no significant difference in the proportion of mortality between the two groups Table 3.

**Table 3:** presents the outcome of TC vs SL in patients who have undergone PDA closure in the Cardiac Center of Ethiopia, 2012-2023

Outcome	TC	SL	P value
Length of mechanical ventilation median (IQR)(hr.)	4(3-6.75)	9(4-13)	0.001
Length of ICU stay median (IQR)(days)	1(1-3)	3(1-5)	0.001
Length of hospital stay median (IQR)(days)	2(2-2.1)	4(2-6)	0.001
Failed device closure n (%)	6(1.9%)	-	-
small residual PDA	34(10.8)	68(19.5)	0.01
Moderate residual PDA	4(1.3)	12(3.4)	0.02
Death n (%)	1(0.3)	0	0.99

## Discussion

Our study compares the immediate outcomes of SL and TC closure of PDA in a cardiac center in Ethiopia. Our finding showed that the SL group had statistically significantly greater percentages of overall complications, mild LV dysfunction, mild pericardial effusion, systemic hypertension, and a higher need for opioids as a complication. Regarding the presence of residual PDA and hospital stays, our study also revealed that the SL group experienced milder and more moderate residual PDA and longer ICU and hospital stays. In addition, we reported a higher percentage of flow acceleration in RPA and the descending aorta in the TC group.

Similar studies from China, Brazil, and Nigeria corroborated this study's finding that there were more overall complications in the SL group compared to the TC group(10–12). Contrary to this, however, one study from the USA found that TC complications were higher than SL complications(13). Additionally, research from Indonesia revealed no differences in complications magnitude between the two groups(9). The age difference between study participants and the experience of professionals in various nations can both be used to explain the discrepancy in reporting the magnitude of complications between SL and TC.

The higher LV dysfunction percentage in SL found in our study is supported by a previous study that was carried out in Ethiopia, where 42.9% of patients experienced mild LV dysfunction after surgery, as opposed to studies done in Korea and India, where only 25% and 18.6% of patients experienced the same complication(14–16). The decrease in LV preload and rise in LV afterload following PDA ligation account for the decline in LV function. Because PDA causes a left-to-right shunt and increases LV preload after ligation, the LV preload decreases noticeably. The increase in afterload is caused by the fact that, before ligation, the LV pumps blood to both the high-resistant aorta and low-resistance pulmonary circulation. However, after closure, the LV only pumped blood against the high-resistance aorta. Further study is needed since it is unknown why surgical ligation causes LV dysfunction to develop more frequently than TC.

Only a few studies in device closure but none in surgical ligation demonstrated flow acceleration in the RPA. In comparison to studies conducted in Pakistan and South Africa, which revealed flow acceleration in the RPA of 2% and 3.1%, respectively, our study found a comparable figure (1.9%)(17,18). Due to the RPA's anatomical distance from the PDA, the rarity of flow acceleration in RPA following surgical closure can be explained. Even though the RPA is anatomically far away from the PDA, it happens frequently during device closure that the device may embolize to the RPA.

Acceleration of blood flow in the descending aorta as a result of device embolization or encroachment of the device to the descending aorta, as in our study, has been documented in numerous studies. It has been reported in studies in the USA(1.9%) and (0.3%)(19), Saudi Arabia (7.4%), Sudan (0.7%), Egypt (0.3%), and in our study (2.5%)(20–23). After surgical PDA ligation, there is a possible risk of inadvertent injury to the descending aorta (24). But reports are very rare.

Even though PDA is an extracardiac structure, there have been reports of pericardial injury and pericardial effusion in other studies, such as those conducted in the UK and Finland, with pericardial effusion proportions of 0.3% and 1%, respectively, which are lower than the 3.3% described in our study(25,26). This variation in pericardial effusion prevalence following surgical PDA ligation can be attributed to the surgeon's experience. In our research, there were only 2 patients in the device group who experienced pericardial effusion; nevertheless, there have been no reports of pericardial effusion following device closure of PDA. This can be a result of the chest device technique not involving any external manipulation.

A study from Korea showed a significant number of patients developed systemic hypertension after PDA surgical ligation (27). Our report of 11.5% systemic hypertension following PDA ligation is higher than that reported from a study in Canada (4.7%) but lower than that from a study in Brazil (67%)(12,28). A possible cause is sympathetic nervous system hyperactivity, which led to a significant increase in catecholamines. There are limited reports of systemic hypertension following TC; one study from Nigeria found 6.3% of systemic hypertension following device closure, while Brazilian research did not find any cases of systemic hypertension(10)(12). Therefore, the manipulation of the aortic arch that enhances the sympathetic surge is high in SL compared to TC, which can be used to explain why there is a higher prevalence of systemic hypertension following SL.

Comparable to our study, Patients in a Brazilian comparison study who underwent surgical ligation needed more opioids than those who underwent transcatheter closure (12). This can be explained by the fact that the expected pain score is high for surgical ligation because it is more invasive than TC.

Similar to a study conducted in Pakistan, our research found that the surgical group had more residual PDA than the TC group did (8). However, research from the USA and China disputes our findings (13)(11). Furthermore, in contrast to our data, another Chinese study found no variation in the magnitude of residual in the TC and SC (29). This discrepancy in residual PDA reporting may be caused by the fact that surgical expertise and interventional cardiology ability are the key determinants of residual PDA occurrence.

Studies from the United States, Brazil, Sudan, China, Indonesia, and Pakistan corroborated our study's findings that patients who underwent surgical ligation had longer hospital stays and stays in the intensive care unit (8,9,12,21,30). The overall complications are more prevalent in SL of PDA than TC of PDA, which explains this.

TC of PDA has fewer overall complications, shorter hospital stays, fewer stays in the intensive care unit, and fewer stays on mechanical ventilation. However, TC of PDA has more complications in major vessels such as flow acceleration in the arch, LPA, RPA, and absence of a pulse in the femoral artery. Given the lower number of overall complications, shorter hospital stays, fewer stays in the intensive care unit, and lower number of days on mechanical ventilation, TC of PDA has to be considered for selected patients.

Transcatheter Closure (TC) of Patent Ductus Arteriosus (PDA) presents several advantages over surgical closure, including fewer overall complications, shorter hospital stays, fewer stays in the intensive care unit (ICU), and reduced days on mechanical ventilation. However, it's crucial to acknowledge that TC of PDA may entail certain specific complications related to major vessels, such as flow acceleration in the aortic arch, left pulmonary artery (LPA), right pulmonary artery (RPA), and potential absence of a pulse in the femoral artery. Considering the substantial benefits in terms of reduced complications and shorter hospital stays, TC of PDA should be strongly considered for carefully selected patients.

This study has few limitations. The cross-sectional nature of the study design may lead to limitations in identifying complications associated with TC and SL closure of PDA that can be detected in the medium to long term, and its retrospective nature can lead to classification bias. Additionally, because this was a single-center study, generalizations cannot be made. However, this study is important for improving the quality of TC and SL closure of PDA in Ethiopia because it provides important baseline data facilitates comparison of the centers' results with those of other centers, and can help make management decisions (select TC or SL PDA for PDA closure) and help establish funding priorities and resource allocation during PDA closure.
